# A novel bispecific antibody dual-targeting approach for enhanced neutralization against fast-evolving SARS-CoV-2 variants

**DOI:** 10.3389/fimmu.2023.1271508

**Published:** 2023-09-26

**Authors:** Ji Woong Kim, Hyun Jung Kim, Kyun Heo, Yoonwoo Lee, Hui Jeong Jang, Ho-Young Lee, Jun Won Park, Yea Bin Cho, Ji Hyun Lee, Ha Gyeong Shin, Ha Rim Yang, Hye Lim Choi, Hyun Bo Shim, Sukmook Lee

**Affiliations:** ^1^ Department of Chemistry, Kookmin University, Seoul, Republic of Korea; ^2^ Department of Biopharmaceutical Chemistry, Kookmin University, Seoul, Republic of Korea; ^3^ Antibody Research Institute, Kookmin University, Seoul, Republic of Korea; ^4^ Department of Nuclear Medicine, Seoul National University Bundang Hospital, Seoul, Republic of Korea; ^5^ Division of Biomedical Convergence, Kangwon National University, Chuncheon, Republic of Korea; ^6^ Department of Life Sciences, Ewha Womans University, Seoul, Republic of Korea

**Keywords:** bispecific antibody, fusion peptide, phage display, receptor-binding domain, SARS-CoV-2

## Abstract

**Introduction:**

The emergence of new severe acute respiratory syndrome coronavirus 2 (SARS-CoV-2) variants has caused unprecedented health and socioeconomic crises, necessitating the immediate development of highly effective neutralizing antibodies. Despite recent advancements in anti-SARS-CoV-2 receptor-binding domain (RBD)-specific monoclonal antibodies (mAbs) derived from convalescent patient samples, their efficacy against emerging variants has been limited. In this study, we present a novel dual-targeting strategy using bispecific antibodies (bsAbs) that specifically recognize both the SARS-CoV-2 RBD and fusion peptide (FP), crucial domains for viral attachment to the host cell membrane and fusion in SARS-CoV-2 infection.

**Methods:**

Using phage display technology, we rapidly isolated FP-specific mAbs from an established human recombinant antibody library, identifying K107.1 with a nanomolar affinity for SARS-CoV-2 FP. Furthermore, we generated K203.A, a new bsAb built in immunoglobulin G4-(single-chain variable fragment)_2_ forms and demonstrating a high manufacturing yield and nanomolar affinity to both the RBD and FP, by fusing K102.1, our previously reported RBD-specific mAb, with K107.1.

**Results:**

Our comprehensive *in vitro* functional analyses revealed that the K203.A bsAb significantly outperformed the parental RBD-specific mAb in terms of neutralization efficacy against SARS-CoV-2 variants. Furthermore, intravenous monotherapy with K203.A demonstrated potent *in vivo* neutralizing activity without significant *in vivo* toxicity in a mouse model infected with a SARS-CoV-2 variant.

**Conclusion:**

These findings present a novel bsAb dual-targeting strategy, directed at SARS-CoV-2 RBD and FP, as an effective approach for rapid development and management against continuously evolving SARS-CoV-2 variants.

## 1 Introduction

The severe acute respiratory syndrome coronavirus 2 (SARS-CoV-2) outbreak that began in late 2019 has caused an unprecedented confluence of global health and socioeconomic crises, making coronavirus disease 2019 (COVID-19) the most devastating viral scourge of the 21st century on a worldwide scale (1, 2). The high transmissibility and rapid mutability of SARS-CoV-2 cause significant morbidity and mortality (3). As of July 2023, an estimated 769 million individuals have succumbed to SARS-CoV-2 infection, resulting in over 6.9 million confirmed fatalities across the globe (4). The clinical manifestations of SARS-CoV-2 infection have been diverse, showing a wide range of severity among those infected. While many individuals experience mild symptoms or remain asymptomatic, others suffer from severe illnesses. Respiratory symptoms are common, and in severe cases, acute respiratory distress syndrome can occur, leading to fatal respiratory failure (5). Despite the recent advancement of multiple neutralizing antibodies, the ongoing emergence of novel SARS-CoV-2 variants has rendered these therapeutics less effective, necessitating the prompt development of interventions with heightened efficacy (6, 7). Therefore, it is imperative to unravel the underlying pathological mechanisms governing SARS-CoV-2 infection and develop novel approaches that more effectively modulate viral propagation.

SARS-CoV-2, a member of the *Betacoronavirus* genus, is an enveloped, single-stranded, positive-sense RNA virus that consists of four essential proteins, including spike (S), envelope, membrane, and nucleocapsid proteins (8). The spike protein, which consists of the functional subunits S1 and S2, is of utmost significance (9). Notably, the RBD located within the S1 subunit plays a pivotal role in viral entry through direct interaction with the human angiotensin converting enzyme 2 (hACE2) receptor on the surface of host cells (10). Leveraging the significance of this interaction, several RBD-specific neutralizing antibodies, including tixagevimab and cilgavimab by AstraZeneca, sotrovimab by GSK, regdanvimab by Celltrion, and casirivimab and imdevimab by Eli Lilly, were developed and received US FDA approval (11–14). Subsequent to RBD-hACE2 binding, host cell proteases cleave the S1/S2 site, triggering a conformational change that exposes the fusion peptide (FP) within the S2 subunit (15). The virus membrane and the host cell’s membrane fuse as a result of the exposed FP, facilitating the entry of viral genetic material into the host cell and subsequent viral replication (16). Dacon et al. recently introduced monoclonal antibodies (mAbs) that specifically target the FP, namely COV44-62 and COV44-79, shedding light on the potential of the FP as a crucial target for the broad neutralization of SARS-CoV-2 variants (17).

Bispecific antibodies (bsAbs) represent a distinctive class of antibodies with two antigen-binding sites that can specifically bind two different antigens or two diverse epitopes on the same antigen (18). This unique feature confers significant advantages by potentiating therapeutic efficacy without necessitating the production of additional mAbs used in combination therapies (19). As of July 2023, the US FDA had approved nine bsAbs to treat various diseases, from hemophilia A to tumor malignancies, while more than 200 bsAbs are undergoing clinical investigation (20, 21). While bsAbs have predominantly been applied in cancer immunotherapy, combination mAb therapies have been developed to combat infectious diseases, bolstering therapeutic effectiveness (22, 23). For instance, the combination of mAbs VRC07-523LS, PGT121, and PGDM1400 has shown promising results in neutralizing diverse HIV-1 strains and is currently undergoing phase I/IIa clinical trials (24). Similarly, the FDA has authorized two antibody cocktails, tixagevimab/cilgavimab and casirivimab/imdevimab, for clinical use in COVID-19 treatment (11, 14). Nonetheless, mAb cocktail approaches have limitations, including heightened manufacturing costs, time-consuming processes, and high-dose infusions for patients (25, 26). Thus, bsAbs emerge as a promising strategy to confront ever-evolving SARS-CoV-2 variants.

In this study, we present a pioneering approach to bsAb development with the potential to timely and efficiently manage SARS-CoV-2 variants. Our methodology leverages phage display technology and an established human recombinant antibody library to rapidly generate a human bsAb, providing a proof of concept for our approach. Additionally, we propose for the first time, the dual-targeting of the SARS-CoV-2 RBD and FP, two pivotal domains for viral attachment to host cell membrane and fusion in SARS-CoV-2 infection, is a promising platform to enhance SARS-CoV-2 neutralization. Substantiated by comprehensive *in vitro* and *in vivo* virological assessments, our approach exhibits broad and enhanced efficacy against diverse SARS-CoV-2 variants. Therefore, our novel bsAb strategy may hold vast potential for the prompt and effective management of the incessantly adapting SARS-CoV-2.

## 2 Materials and methods

### 2.1 Cell culture

HEK293T, Vero E6, K562, and THP-1 cells were obtained from the American Type Culture Collection (ATCC, Rockville, MD, USA), and Expi293F cells were procured from Thermo Fisher Scientific (Waltham, MA, USA). Dulbecco’s modified Eagle medium (DMEM, Thermo Fisher Scientific) was used for HEK293T and VeroE6 cells, while Roswell Park Memorial Institute (RPMI, Thermo Fisher Scientific) media was used for K562 and THP-1 cells. Both media were supplemented with 10% (v/v) fetal bovine serum (FBS, Thermo Fisher Scientific) and 100 U/mL penicillin–streptomycin (Thermo Fisher Scientific). The cells were maintained at 37°C with 5% CO_2_. Expi293F cells were cultured in Expi293™ Expression Media in shaking incubators at 37°C, 125 rpm, and 8% CO_2_. Cell counting was performed using ADAM™ CellT (NanoEntek, Seoul, Republic of Korea).

### 2.2 Peptide synthesis

The SARS-CoV-2 FP peptide (SFIEDLLFNKVTLADAGF) with a cysteine residue at the C-terminus was chemically synthesized and conjugated to bovine serum albumin (BSA) or ovalbumin (OVA) by Peptron (Daejeon, Republic of Korea). Reverse-phase high-performance liquid chromatography (HPLC) was performed to purify the peptide conjugates using a Vydac Everest C18 column (250 mm × 22 mm, 10 μm; Uvison, Sevenoaks, United Kingdom). The purity of all synthesized peptides was confirmed to be greater than 95%, and their molecular mass was verified by liquid chromatography–mass spectrometry using an Agilent HP1100 series HPLC system (Santa Clara, CA, United States).

### 2.3 Isolation of SARS-CoV-2 FP-specific human antibodies

Biopanning was performed from a human recombinant single-chain variable fragment (scFv) library using phage display technology to isolate SARS-CoV-2 FP-specific human mAbs. Four rounds of biopanning were carried out with M-270 epoxy Dynabeads™ (Invitrogen, Carlsbad, CA, USA), which were covalently coupled with 4 μg of either FP-BSA or FP-OVA. The first and third rounds of biopanning used FP-BSA-conjugated beads, and the second and fourth rounds used FP-OVA-conjugated beads to select FP-specific human scFv antibody clones. After completing the four rounds of biopanning, randomly selected 96 phage clones from the output colonies were then subjected to a phage enzyme-linked immunosorbent assay (ELISA) to test their reactivity to target antigens. Following DNA sequencing, scFv antibody clones with unique complementarity-determining region (CDR) sequences were chosen for further study.

### 2.4 Phage ELISA

To identify binders specific to SARS-CoV-2 FP, each colony from the 4^th^ round of biopanning was cultured in 1 mL of Super Broth (SB) media with 50 μg/mL of carbenicillin in 96-deep-well plates (Axygen, Union City, CA, USA) at 37°C overnight. Then, 10^10^ plaque-forming units (PFU) of helper phages (VCSM13; Agilent, Santa Clara, CA, USA) were added into each well, followed by the addition of kanamycin to the media with a final concentration of 70 μg/mL, and the mixture was incubated overnight at 37°C. The supernatant was obtained by centrifugation at 3,000 g and used for phage ELISA. High-binding 96-well microplates (Corning, Corning, NY, USA) were coated overnight with 0.1 μg of either FP-BSA or FP-OVA in phosphate-buffered saline (PBS) at 4°C. After blocking with 3% (w/v) BSA in PBS, the plates were incubated with 100 μL of phage supernatant at 37°C for 2 h. Subsequently, the plates were washed three times with PBS containing 0.05% (v/v) Tween 20 (PBST). HRP-conjugated anti-hemagglutinin antibody (1:3,000; Bethyl Laboratories, Montgomery, TX, US) was added and incubated at 37°C for 1 h. For colorimetric detection, 3,3’,5,5’-tetramethylbenzidine (TMB) substrate solution (Thermo Fisher Scientific) was added to the wells. The reactions were stopped by adding 1 M of H_2_SO_4_, and the absorbance was measured at 450 nm using a microtiter plate reader (Bio-Tek Instruments, Winooski, VT, USA).

### 2.5 Construction, expression, and purification of selected antibodies

The selected scFv clones were cloned into an IgG4-encoding pCEP4 mammalian expression vector (Thermo Fisher Scientific) to generate scFv-Fc antibodies. Here, IgG4 was engineered to contain an S228P mutation to prevent IgG4 Fab-arm exchange. Following biochemical and functional characterization, an scFv clone with strong affinity and a high production yield was designated as K107.1 and used as the parental antibody for bsAb generation.

We designed three types of IgG4-(scFv)_2_ fusion proteins by fusing the scFv region of K107.1 with K102.1, an anti-SARS-CoV-2 RBD-specific IgG4 with an S228P mutation, to create bsAbs. The scFv was linked to the C-terminus of the heavy chain of K102.1 (type A), the C-terminus of the light chain of K102.1 (type B), or the N-terminus of the light chain of K102.1 (type C). Each recombinant DNA was transiently expressed using the Expi293 Expression System (Thermo Fisher Scientific) following the manufacturer’s recommendations. The overproduced antibodies were purified from the culture media using affinity column chromatography with Protein A-Sepharose (Repligen, Waltham, MA, USA) as previously described (27).

### 2.6 Surface plasmon resonance

The real-time interaction between antibodies and antigens was assessed using surface plasmon resonance (SPR) with a Biacore T200 instrument (Cytiva, Marlborough, MA, USA). The CM5 sensor chip (Cytiva) was prepared by activating its carboxyl groups using a mixture of 1-ethyl-3-(3-dimethylaminopropyl) carbodiimide and N-hydroxysuccinimide. Recombinant wild-type SARS-CoV-2 RBD or FP-BSA was then covalently immobilized on the sensor chip surface for the immobilization of 250 response units through standard amine coupling. To evaluate the binding interaction, FP-specific antibodies (K107.1, K107.2, and K107.3) were injected onto the sensor chip with immobilized FP-BSA, and increasing concentrations of the antibodies (8, 16, 32, 64, and 128 nM) were used at a flow rate of 50 µL/min. Similarly, the bsAb was injected onto the sensor chip containing either immobilized wild-type SARS-CoV-2 RBD or FP-BSA at a flow rate of 50 µl/min. After each binding cycle, the sensor chips were regenerated by injecting 10 mM glycine-HCl (pH 3.0), which effectively removed any bound antibodies from the sensor chip surface. The equilibrium dissociation constants (K_D_) were determined using Biacore T200 Control Software Version 3.2 (Cytiva).

To further verify the properties of K203.A bsAb and its independent binding to different target sites, additional real-time binding experiments were conducted. In this experiment, FP-BSA or RBD was first immobilized on the CM5 sensor chip surface. Then, 1 μM of K203.A was introduced onto the sensor chip surface to saturate the binding sites corresponding to the FP. Next, purified SARS-CoV-2 RBD or FP-BSA was introduced onto the same sensor chip with the K203.A-saturated surface. The generated binding curves were analyzed using Prism Software 8.0 (GraphPad Software, La Jolla, CA, USA).

### 2.7 Protein thermal shift assay

A protein thermal shift assay was conducted to investigate the thermal stability of the K203.A antibody. In each well of a MicroAmp^®^ Optical 8-tube strip (Applied Biosystems, Foster City, CA, USA), 5 μg of K203.A antibody was added. Additionally, 2.5 μL of Protein Thermal Shift Dye (8×, Applied Biosystems, Foster City, CA, USA) was added. For the negative control, PBS was mixed with Protein Thermal Shift Dye. The thermal shift measurements were performed using a real-time PCR instrument (QuantStudio™ 3 Real-Time PCR, Applied Biosystems), according to the manufacturer’s instructions. Furthermore, all experiments were performed in duplicate to ensure reliability and accuracy.

### 2.8 SARS-CoV-2 RBD-human ACE2 neutralization assay

The inhibitory potency of selected antibodies on the interaction between the purified SARS-CoV-2 RBD and hACE2 was evaluated using ELISA. Fc-tagged hACE2 (hACE2-Fc; R&D Systems, Minneapolis, MN, USA) was immobilized on 96-well plates at a concentration of 50 ng per well. After blocking with a blocking buffer (BPS Bioscience), 25 nM of purified His-tagged SARS-CoV-2 wild-type or Delta variant RBD (Sino Biological) was preincubated with either K102.1 mAb or K203.A bsAb at concentrations of 1 nM or 10 nM for 1 h at 25°C. The preincubated mixtures of RBD and antibodies were then added to the wells containing immobilized hACE2-Fc and incubated for an additional 1 h. An HRP-conjugated anti-His secondary antibody (BPS Bioscience) was used for detection. Furthermore, the neutralizing activity of the selected antibodies against the RBD-hACE2 interaction was assessed by measuring chemiluminescence intensity with a Synergy H1 microplate reader (Bio-Tek Instruments).

### 2.9 SARS-CoV-2 pseudovirus neutralization assay

Replication-deficient Moloney murine leukemia virus (MLV) particles were used to express variants of the SARS-CoV-2 S protein and a firefly luciferase reporter gene. The MLV particles were obtained from eEnzyme (Gaithersburg, MD, USA). HEK293T/hACE2 cells (1 × 10^4^ cells in 50 μL culture medium) were seeded in 96-well tissue culture plates and allowed to adhere overnight. The following day, serially diluted K102.1 mAb, K203.A bsAb, or K107.1 mAb at a 200 nM concentration were each preincubated with pseudovirus (1 × 10^7^ PFU/mL) for 10 min at 25°C to allow interaction with the virus. After the preincubation, the antibody–pseudovirus mixtures were added to the cells and incubated for 24 h. To assess viral infection, the presence of the firefly luciferase reporter gene was assessed using ONE-Glo™ luciferase substrate (Promega, Madison, WI, USA). In addition, luminescence signals were measured with a Synergy H1 microplate reader (Bio-Tek Instruments), and the half-maximal inhibitory concentration (IC_50_) values were determined using Prism Software 8.0 (GraphPad Software).

### 2.10 SARS-CoV-2 live virus neutralization assay

SARS-CoV-2 wild-type (BetaCoV/Korea/KCDC03/2020, NCCP no. 43326) and Delta variant (hCoV-19/Korea/KDCA119861/2021, NCCP no. 43390) were obtained from the Korea Disease Control and Prevention Agency (KDCA, Osong, Republic of Korea). These viruses were propagated in Vero E6 cells and titrated using a plaque assay. Subsequently, they were stored at −80°C for subsequent *in vitro* infection assessments. For the *in vitro* infection assessment, VeroE6 cells (1.5 × 10^4^ cells in 100 μL of culture medium) were seeded on 96-well tissue culture plates and allowed to adhere overnight. Serial dilutions of the antibodies were preincubated with SARS-CoV-2 (at a concentration of 4 × 10^2^ TCID_50_/mL) for 1 h at 25°C to allow the interaction of antibodies with the virus. After the preincubation period, the virus/antibody mixture was added to the Vero E6 cells. The cells were then incubated with the virus/antibody mixture for three days. For the determination of neutralization potency, reverse transcription-quantitative polymerase chain reaction (RT-qPCR) was performed for viral RNA quantification using the QuantiTect SYBR^®^ Green RT-PCR kit (Qiagen, Hilden, Germany).

### 2.11 *In vivo* mouse study

For *in vivo* efficacy studies, 8-week-old male B6.Cg-Tg(K18-ACE2)2Prlmn/J mice (The Jackson Laboratory, CA, USA) were used. These mice were transgenic for hACE2 and served as a model for studying SARS-CoV-2 infection. The mice were housed in a certified animal biosafety level 3 (ABSL3) facility at the Ji Seok-Yeong Biomedical Research Institute of Seoul National University Bundang Hospital in Seongnam, Republic of Korea. All experimental procedures were ethically reviewed and approved by the Institutional Animal Care and Use Committee (IACUC; Approval No. BA-2108-325-078). Additionally, the Institutional Biosafety Committee of Seoul National University Bundang Hospital approved biosafety experimental protocols (Approval No. IBC-2105-A-008) to ensure the safety of researchers and animals during the study. The hACE2-transgenic (TG) mice (*n* = 4) were intranasally inoculated with 50 μL of Delta variant virus (1 × 10^4^ PFU) under anesthesia. After three hours of infection, either PBS, 5 mg/kg or 30 mg/kg of K203.A bsAb was intravenously injected. Six days after the SARS-CoV-2 Delta variant infection, lung tissues were harvested from the hACE2-TG mice. Furthermore, the viral RNA present in the lung tissues was quantified using RT-qPCR.

### 2.12 Histology

The mice were euthanized by CO_2_ as per approved animal welfare guidelines. The lung tissues were then collected and fixed in 10% neutral buffered formalin (Sigma, St. Louis, MO, USA) for 24 h. Furthermore, the lung tissues were processed for paraffin embedding. In this procedure, the tissues were dehydrated using a series of alcohol solutions and then infiltrated with paraffin wax. The paraffin-embedded tissues were then sectioned at a 4 μm thickness using a microtome. The sections were subjected to hematoxylin and eosin staining. Histopathological examination was performed using light microscopy (Olympus, Tokyo, Japan).

### 2.13 *In vivo* toxicity and pharmacokinetic analysis

For the *in vivo* toxicity and serum pharmacokinetic analysis, 8-week-old female Institute of Cancer Research (ICR) mice (Orient Bio Inc., Seongnam, Republic of Korea) were used. The study received approval from the Institutional Animal Care and Use Committee (IACUC) of the National Cancer Center, Republic of Korea (Approval No. NCC-21-693). A total of three mice were included in the study. They received an intravenous injection of either 5 mg/kg or 30 mg/kg of K203.A bsAb. The body weight of each mouse was monitored regularly for 21 days post-injection to evaluate the potential systemic toxicity of K203.A. At the end of the 21-day period, blood and serum samples were obtained from the mice. The levels of several biochemical markers, including glutamic oxaloacetic transaminase (GOT), glutamic pyruvic transaminase (GPT), total bilirubin (TBIL), creatinine (CRE), and blood urea nitrogen (BUN), were quantified from these serum samples using a Fuji Dri-Chem 3500 Biochemistry Analyzer (Fujifilm, Tokyo, Japan).

To determine the pharmacokinetic profile of K203.A, 50 µL of blood samples were collected from each mouse at specific time points: 4, 8, 24, 72, 120, 168, 264, 384, and 504 h post-injection. The blood samples were centrifuged at 5,000 × g for 20 minutes at 4°C for the collection of the serum samples. The K203.A concentration in the serum samples was measured at each time point using a human IgG ELISA kit (Abcam) based on the manufacturer’s instructions. Optical density values were measured at 450 nm using a Synergy H1 microplate reader (Bio-Tek Instruments).

### 2.14 *In vitro* antibody-dependent enhancement assay

To investigate antibody-dependent enhancement (ADE), HEK293T, HEK293T/hACE2, K562, or THP-1 cells (1 × 10^4^ cells/well) were seeded in 96-well plates. Different concentrations of K203.A bsAb (0.044 to 100 nM) were preincubated with each variant of the SARS-CoV-2 pseudovirus (wild-type, D614G, Alpha, Beta, Gamma, Delta, and Kappa; 1 × 10^7^ PFU/mL) for 30 min at 25°C. The mixtures of bsAb and pseudoviruses were added to the respective cells in the 96-well plates, which were then cultured for 24 h. To assess the level of viral infection in the cells, luciferase activity was measured as described in Section 2.8.

### 2.15 Statistical analysis

The statistical analysis of the data was performed using Prism Software 8.0 (GraphPad Software). A two-tailed Student’s t-test was used when comparing data between two groups, and a one-way analysis of variance (ANOVA) with Bonferroni’s correction was used for multiple comparisons between more than two groups. Results are expressed as the mean ± standard deviation (SD). Statistical significance was considered at *P* < 0.05 (*), *P* < 0.01 (**), and *P* < 0.001 (***).

## 3 Results

### 3.1 Isolation of phage display-derived human antibodies specific to SARS-CoV-2 FP

**Figure 1:**
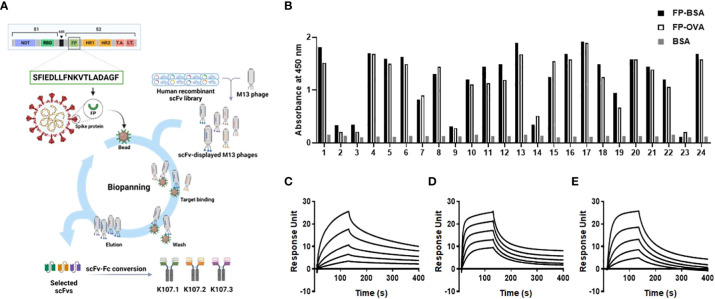
Isolation and characterization of SARS-CoV-2 FP-specific antibodies. **(A)** Schematic representation of the selection strategy for FP-specific antibodies using phage display technology from a human recombinant scFv library. **(B)** The binding specificity of randomly selected clones against SARS-CoV-2 FP was confirmed using phage ELISA after four rounds of biopanning. BSA served as a negative control. The affinities of the selected antibodies to SARS-CoV-2 FP were measured using SPR. The sensorgram from each SPR experiment represents the interaction of different concentrations (8, 16, 32, 64, and 128 nM) of the following antibodies to FP-BSA immobilized on the sensor chip: **(C)** K107.1; **(D)** K107.2; and **(E)** K107.3. The y-axis of the SPR sensorgrams represents the RU, a measure of the antibody’s binding to the immobilized FP-BSA. The x-axis denotes time in seconds. These measurements allowed the determination of kinetic parameters, leading to the calculation of the K_D_ values for each antibody against the SARS-CoV-2 FP. Fusion peptide, FP; single-chain variable fragment, scFv; enzyme-linked immunosorbent assay, ELISA; surface plasmon resonance, SPR; response unit, RU; equilibrium dissociation constant, K_D_.

We employed biopanning, a powerful approach based on phage display technology (27), to isolate human antibodies that target the SARS-CoV-2 FP. Using this iterative technique, we subjected a well-established human recombinant scFv library containing SARS-CoV-2 FP-specific scFvs to a series of selection rounds (**Figure 1A**). During each round, we exposed the scFv library to immobilized FP-BSA and FP-OVA, allowing the scFvs with binding affinity to the target FP to interact with and attach to the solid support. Unbound scFvs were subsequently washed away to eliminate non-specific binders, while the specifically bound scFvs were eluted and amplified using host bacteria for subsequent rounds of selection.

A phage ELISA assay showed that we isolated a variety of scFv clones with strong binding affinity to FP-BSA and FP-OVA following numerous rounds of biopanning (**Figure 1B**). Importantly, these scFv clones did not bind to BSA, which was used as a negative control, thus confirming their specificity for the SARS-CoV-2 FP. The biopanning process served as an effective and robust method for enriching the human antibody library with scFvs that specifically recognize the SARS-CoV-2 FP.

To further refine our selection, we chose three scFv clones with distinct complementarity-determining region sequences, all of which exhibited specific binding and strong affinity to the SARS-CoV-2 FP. Subsequently, we transformed these scFv clones into human scFv-Fc antibodies by fusing the scFvs to the Fc region of human immunoglobulin G (IgG). These recombinant human scFv-Fc antibodies were produced using a suspension-adapted HEK293 cell line (Expi293F), an expression system optimized for large-scale antibody production. Furthermore, we purified antibodies using Protein A-Sepharose affinity chromatography. The purity of these antibodies exceeded 90%, as shown in **Supplementary Figure 1**, and the production yields were approximately 50 mg/L, 30 mg/L, and 80 mg/L, respectively, as illustrated in **Supplementary Figure 2**.

To evaluate the binding kinetics between the chosen antibodies and SARS-CoV-2 FP, we conducted real-time kinetic analysis and determined the dissociation constant (K_D_) values for K107.1, K107.2, and K107.3 against SARS-CoV-2 FP as 7.18 nM, 17.29 nM, and 34.57 nM, respectively (**Figures 1C–E** and **Table 1**). Among these antibodies, K107.1 exhibited the most remarkable binding affinity towards the SARS-CoV-2 FP, suggesting a strong and specific interaction. Based on these results, we chose K107.1 as the parental antibody specific to the FP.

**Table 1:** Measurement of the binding kinetics of the selected scFv-Fc antibodies to SARS-CoV-2 FP.

Antibody	^1^K_D_ (nM)	^2^K_a_ (1/M^-1^s^-1^)	^3^K_d_ (s^-1^)
**K107.1**	7.18	4.22 x 10^5^	3.04 x 10^-3^
**K107.2**	17.29	5.17 x 10^5^	8.94 x 10^-3^
**K107.3**	34.57	2.41 x 10^5^	8.34 x 10^-3^

^1^Equilibrium dissociation constant; ^2^Association constant; ^3^Dissociation constant.

### 3.2 Design, generation, and biochemical characterization of bsAbs

**Figure 2:**
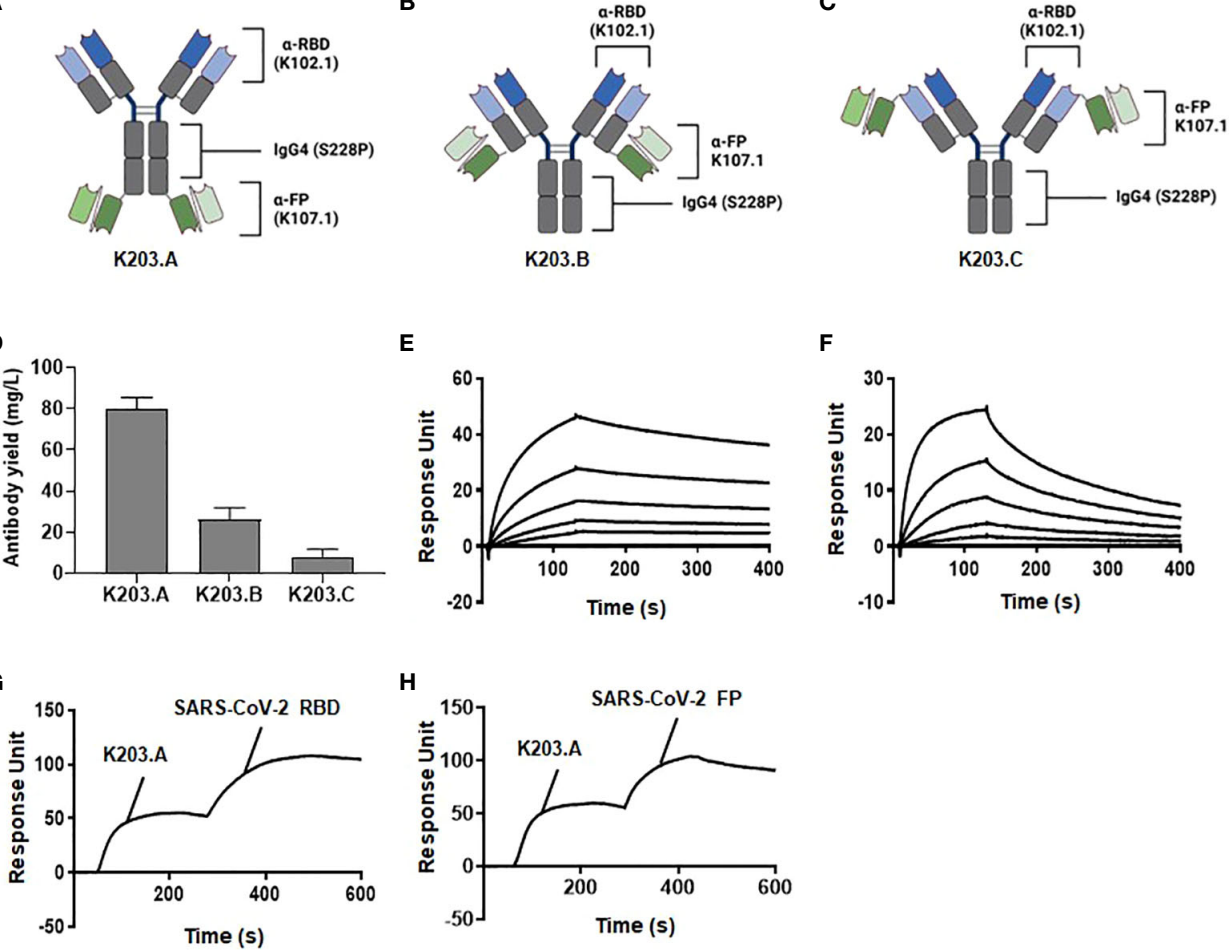
Design, generation, and biochemical characterization of bsAbs. The schematic diagrams of the newly designed and constructed bsAbs in the IgG4-(scFv)_2_ format: **(A)** K203.A; **(B)** K203.B; and **(C)** K203.C. **(D)** Production yield of K203.A, K203.B, and K203.C expressed as mg/L. **(E)** Binding kinetics of K203.A to wild-type SARS-CoV-2 RBD evaluated by SPR analysis. **(F)** Binding kinetics of K203.A to FP-BSA assessed by SPR analysis. **(G)** Additional binding of SARS-CoV-2 RBD on a K203.A-saturated SARS-CoV-2 FP-BSA-immobilized sensor chip, confirming the dual-targeting potential of K203.A. **(H)** Additional binding of FP-BSA on a K203.A-saturated SARS-CoV-2 RBD-immobilized sensor chip, further confirming the dual-targeting potential of K203.A. The data shown represent the findings of two independent experiments. Bispecific antibody, bsAb; receptor-binding domain, RBD; fusion peptide, FP; bovine serum albumin, BSA; surface plasmon resonance, SPR.

Three distinct bsAbs utilizing the IgG4-(scFv)_2_ format were designed to target the RBD and FP of SARS-CoV-2. These were generated based on two key components: the previously identified SARS-CoV-2 RBD-specific neutralizing mAb, K102.1, and the newly discovered FP-specific antibody, K107.1 (27). The three types of bsAbs, designated as K203.A, K203.B, and K203.C, were engineered to optimize their binding properties (**Figures 2A–C**). K203.A was designed with scFvs attached to the C-terminus of each IgG4 heavy chain. In K203.B, the scFvs were linked to the C-terminus of the IgG4 light chains, while K203.C was constructed with scFvs connected to the N-terminus of each IgG4 light chain.

We incorporated the S228P mutation into the IgG4 region of each bsAb to ensure their stability and homogeneity. This mutation was strategically introduced to prevent unwanted Fab arm exchange, which could lead to heterogeneous antibody mixtures due to half-molecule exchange with endogenous IgG4 (28). By adopting the IgG4-(scFv)_2_ format and incorporating the S228P mutation, we aimed to create bsAbs with improved stability and specific binding capacity to both the RBD and FP of SARS-CoV-2.

Upon successful overproduction and purification, the final production yields of purified K203.A, K203.B, and K203.C were approximately 80, 26, and 8 mg/L, respectively (**Figure 2D**). The purity of the resulting bsAbs was over 90%, verified through sodium dodecyl sulfate polyacrylamide gel electrophoresis (SDS-PAGE) and Coomassie Brilliant Blue staining (**Supplementary Figure 3**). Given the importance of production yield in antibody development, we selected K203.A, which had a superior production yield, as the prime bsAb for additional studies.

To assess the binding affinity of K203.A to both SARS-CoV-2 RBD and FP, we conducted real-time SPR analysis using wild-type SARS-CoV-2 RBD and FP-BSA as binding partners. The obtained K_D_ values for K203.A against SARS-CoV-2 RBD and FP were 1.65 nM and 8.76 nM, respectively (**Figures 2E, F**, and **Table 2**). Moreover, we evaluated the thermal stability of K203.A through a protein thermal shift (PTS) assay, which measures the melting temperature (T_m_) of the antibody. K203.A exhibited robust thermal stability, with a T_m_ value of 72.03°C (**Supplementary Figure 4**).

**Table 2:** Measurement of the binding kinetics of K203.A to wild-type SARS-CoV-2 RBD and FP.

Antigen	^1^K_D_ (nM)	^2^K_a_ (1/M^-1^s^-1^)	^3^K_d_ (s^-1^)
**SARS-CoV-2 RBD**	1.65	3.31 x 10^5^	5.46 x 10^-4^
**SARS-CoV-2 FP**	8.76	4.36 x 10^5^	3.82 x 10^-3^

^1^Equilibrium dissociation constant; ^2^Association constant; ^3^Dissociation constant.

To corroborate and further solidify the binding affinity of K203.A towards both SARS-CoV-2 RBD and FP, we performed additional experiments using SPR. We utilized sensor chips immobilized with SARS-CoV-2 FP-BSA or SARS-CoV-2 RBD, and first saturated them with K203.A. Subsequently, we introduced additional SARS-CoV-2 RBD or FP to each sensor chip. The results demonstrated the subsequent binding of each applied antigen on both K203.A-saturated sensor chips (**Figures 2G, H**), confirming the dual-targeting potential of K203.A to simultaneously engage with both SARS-CoV-2 RBD and FP.

### 3.3 *In vitro* neutralization potency of K203.A against multiple SARS-CoV-2 variants

To determine the inhibitory activity of K203.A bsAb on the interaction between SARS-CoV-2 RBD and hACE2, we carried out interaction-inhibition assays using ELISA with recombinant hACE2 and RBDs from both the wild-type SARS-CoV-2 and the Delta variant. The assays were conducted in the absence and presence of the parental RBD-specific mAb K102.1 and K203.A. The results demonstrated that K203.A exerted a comparable inhibitory effect on the hACE2–RBD interactions as K102.1 (**Supplementary Figure 5**).

**Figure 3:**
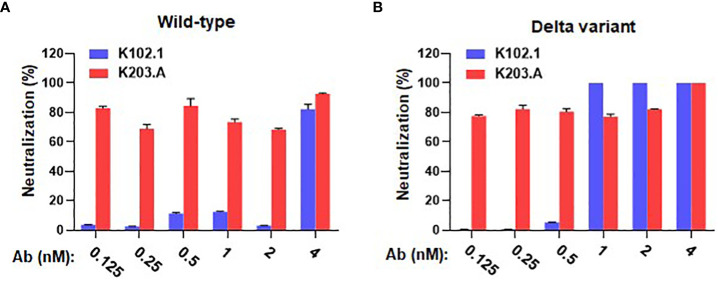
Evaluation of *in vitro* neutralizing activity of K102.1 and K203.A against live SARS-CoV-2 wild-type and Delta variant. RT-qPCR was used to assess the neutralization activity of K102.1 and K203.A against live SARS-CoV-2 **(A)** wild-type and **(B)** Delta variant through the quantification of viral gene expression. Reverse transcription-quantitative polymerase chain reaction, RT-qPCR.

We conducted live virus neutralization assays with the wild-type and the Delta variant of SARS-CoV-2 using the Vero E6 cell line to comprehensively assess the neutralizing activity of K203.A against live viral infections. To assess the neutralization efficacy of K203.A, we systematically increased the concentration of both K102.1 and K203.A. The neutralization efficacy of K203.A was quantified by measuring the expression of the viral open reading frame 1a (ORF1a) gene, a common target in RNA-based quantification methods for SARS-CoV-2 due to its proven effectiveness in COVID-19 diagnosis (29). To perform this quantification, we employed RT-qPCR, a sensitive and widely used technique for precisely measuring the levels of RNA transcripts in a sample. The results revealed that K203.A exhibited markedly enhanced inhibitory efficacy on the expression of the viral gene in both the wild-type SARS-CoV-2 and the Delta variant, thus surpassing the neutralization efficacy of the parental mAb K102.1 (**Figures 3A, B**).

**Figure 4:**
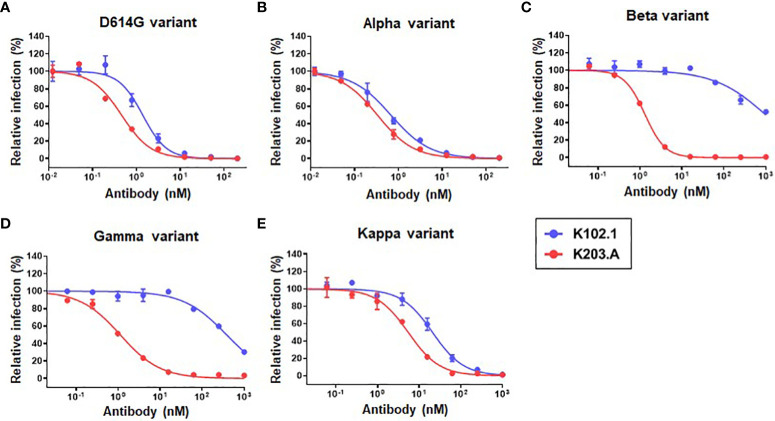
Comparative analysis of the neutralization efficacy of K102.1 and K203.A against SARS-CoV-2 pseudotyped variants. Neutralizing activity of K102.1 (blue) and K203.A (red) against infection with pseudoviral SARS-CoV-2 variants, which include the **(A)** D614G, **(B)** Alpha, **(C)** Beta, **(D)** Gamma, and **(E)** Kappa variants. The neutralizing effect of each antibody was assessed using duplicate measurements from one out of two independent experiments, and the mean values ± standard deviation (SD) are shown. The IC_50_ values, determined through nonlinear regression analysis followed by log (inhibitor) response, provide quantitative measures of the inhibitory effect of the antibodies against the respective variants. Standard deviation, SD; half-maximal inhibition concentration, IC_50_.

To evaluate the neutralization efficacy of K203.A against the SARS-CoV-2 variants, pseudovirus neutralization assays were performed utilizing a HEK293T cell line stably overexpressing hACE2 (HEK293T/hACE2). The assays encompassed several prominent variants, including D614G, Alpha, Beta, Gamma, and Kappa. Throughout the experiments, both K102.1 and K203.A antibodies were present, so their impact on viral infection could be compared. The results demonstrated that K203.A exhibited significantly higher inhibitory effects on infection by all examined pseudovirus types compared to its parental mAb, K102.1. The IC_50_ values for K203.A were in the sub- to low-nanomolar range against all tested variants (**Figures 4A–E** and **Table 3**). On the other hand, the FP-specific mAb K107.1 demonstrated no statistically significant neutralizing efficacy against wild-type SARS-CoV-2 or the D614G, Alpha, Beta, Gamma, Delta, or Kappa variants at 200 nM (**Supplementary Figure 6**). These findings suggest that the development of a bsAb targeting both the SARS-CoV-2 RBD and FP, as exemplified by K203.A, has the potential to achieve enhanced neutralization against diverse SARS-CoV-2 variants.

**Table 3:** IC_50_ values (nM) for K102.1 and K203.

Pseudovirus type	IC_50_ values (nM) for K102.1	IC_50_ values (nM) for K203.A
**D614G**	1.38 ± 0.10	0.42 ± 0.02
**Alpha**	0.67 ± 0.08	0.33 ± 0.02
**Beta**	ND	1.29 ± 0.11
**Gamma**	ND	1.11 ± 0.10
**Kappa**	20.91 ± 1.49	5.46 ± 0.31

Not determined, ND.

A against pseudovirus infection of SARS-CoV-2 variants.

### 3.4 Evaluation of the *in vivo* neutralization efficacy of K203.A against the SARS-CoV-2 Delta variant

**Figure 5:**
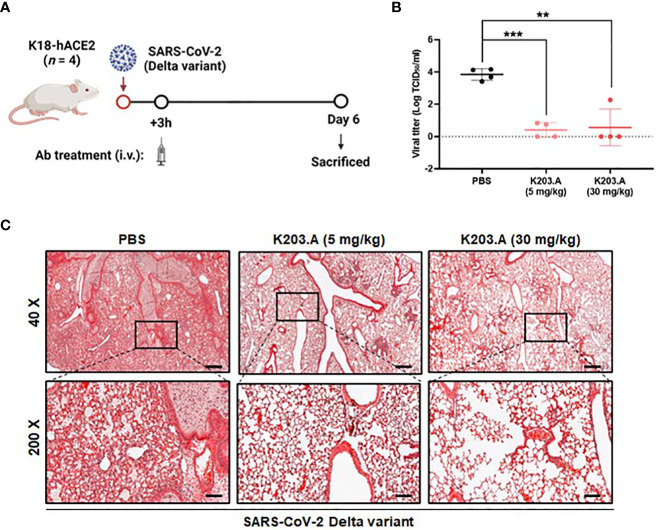
*In vivo* efficacy of K203.A against the SARS-CoV-2 Delta variant in hACE2-TG mice. **(A)** The experimental scheme used for assessing the effectiveness of K203.A against the SARS-CoV-2 Delta variant in hACE2-TG mouse models. **(B)** Viral concentrations in the lung tissues were determined by RT-qPCR at 6 dpi by measuring TCID_50_/mL. **(C)** Histopathological examination: for each group at 6 dpi, representative histopathological pictures of lung tissue are shown. The scale bars at 40× and 200× represent 400 µm and 80 µm, respectively. The data shown are the mean ± SD derived from four biological replicates. Statistical analysis was assessed using a two-tailed Student’s t-test, with **P < 0.01 and ***P < 0.001, indicating significant differences compared with the PBS-treated group. Human angiotensin-converting enzyme 2, hACE2; transgenic, TG; days post-infection, dpi; reverse transcription-quantitative polymerase chain reaction, RT-qPCR; standard deviation, SD; phosphate-buffered saline, PBS.

To determine the *in vivo* neutralization efficacy of K203.A against the SARS-CoV-2 Delta variant, we conducted experiments using K18-hACE2-transgenic (TG) mice that were infected with the virus intranasally. Following a three-hour incubation period, the infected mice were administered two different doses of K203.A through intravenous injection: a low dose of 5 mg/kg and a high dose of 30 mg/kg (**Figure 5A**). At 6 days post-infection (dpi), lung samples were excised from all sacrificed mice, and the viral titer was determined using RT-qPCR. The results revealed a significant reduction in viral titers in both the low-dose and high-dose K203.A-treated groups compared with the PBS-treated groups (**Figure 5B**), highlighting K203.A’s ability to neutralize the virus in the lung tissues of infected mice.

We also conducted a histopathological examination at 6 dpi of the lungs of the mice that were infected with the SARS-CoV-2 Delta variant. The mice that received PBS treatment displayed severe lung damage from the Delta variant infection, with evident pulmonary lesions characterized by pronounced pulmonary edema and alveolar hemorrhage. Conversely, each of the K203.A-treated groups exhibited a marked reduction in pulmonary edema and alveolar hemorrhage, indicating a notable attenuation of lung tissue damage. The histopathological analysis revealed a significant protective effect of K203.A against the deleterious effects of the Delta variant on the lungs (**Figure 5C**). These findings provide strong evidence of the neutralization efficacy of K203.A in mitigating lung pathology caused by the Delta variant infection, highlighting the critical role of bsAbs like K203.A in addressing the challenges posed by evolving SARS-CoV-2 variants.

### 3.5 *In vivo* toxicity assessment and pharmacokinetic analysis of K203.A

**Figure 6:**
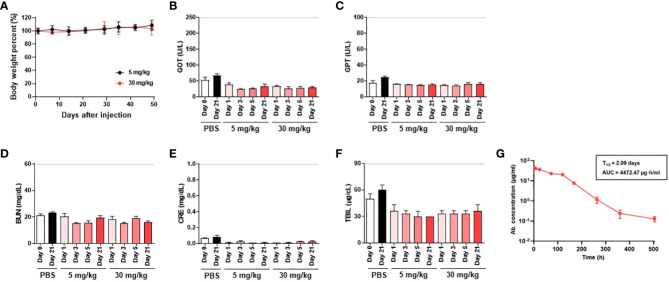
Assessment of *in vivo* toxicity and pharmacokinetics of K203.A. **(A)** Body weight changes of ICR mice administered with two different doses of K203.A (5 mg/kg and 30 mg/kg) were monitored weekly. The *in vivo* toxicity was determined by evaluating alterations in serum concentrations of **(B)** GOT, **(C)** GPT, **(D)** TBIL, **(E)** BUN, and **(F)** CRE gathered from blood samples 0, 1, 3, 5, or 21 days post-administration of PBS or K203.A. The dotted line indicates the safety limit of each enzyme level in mice. **(G)** ELISA was used to analyze serum levels of K203.A from blood samples collected from ICR mice (*n* = 3) intravenously injected with K203.A at a dose of 5 mg/kg. Institute of Cancer Research, ICR; glutamic oxaloacetic transaminase, GOT; glutamic pyruvic transaminase, GPT; total bilirubin, TBIL; creatinine, CRE; blood urea nitrogen, BUN; enzyme-linked immunosorbent assay, ELISA.

An in-depth *in vivo* toxicity study was performed on ICR mice to assess the safety of K203.A. Two different doses of the antibody (5 mg/kg and 30 mg/kg) were administered to the mice through intravenous injections. Throughout the study, we carefully monitored liver and kidney functions, as well as any changes in body weight, to evaluate potential adverse effects. To evaluate liver function, we measured serum concentrations of GOT, GPT, and TBIL. To assess kidney function, we measured BUN and CRE concentrations. The results showed no significant changes in these parameters in the K203.A-treated groups. Thus, the lack of significant alterations in liver and kidney functions, as well as in body weight, suggests the absence of *in vivo* toxicity associated with the administration of K203.A (**Figures 6A–F**).

Following the *in vivo* toxicity assessment, we proceeded with an *in vivo* pharmacokinetic analysis of K203.A to improve our understanding of its behavior in the bloodstream. A 5 mg/kg dose of K203.A was administered to ICR mice, followed by periodic blood sample collection for further examination. The concentration of K203.A in the blood was quantified using ELISA to determine its *in vivo* half-life. In the pharmacokinetic analysis, K203.A showed an *in vivo* half-life of 2.09 days in mice (**Figure 6G**). This pharmacokinetic profile suggests a favorable duration of K203.A’s presence in the bloodstream, which may contribute to its sustained neutralization effect.

To further investigate potential adverse effects, we examined the impact of K203.A on ADE. For this investigation, we employed permissive cells expressing hACE2 (HEK293T/hACE2) and cells expressing the Fc gamma receptor (HEK293T, K562, and THP-1), which were infected with multiple SARS-CoV-2 pseudotyped variants, including wild-type, Alpha, Beta, Gamma, Delta, and Kappa. Notably, no significant changes in pseudovirus infection were observed, indicating that K203.A may not induce ADE (**Supplementary Figure 7**).

Together, these results demonstrate the favorable safety profile of K203.A, as evidenced by the absence of *in vivo* toxicity, its extended half-life, and the lack of antibody-dependent enhancement effects. These findings enhance the potential of the dual-targeting bsAb strategy as a promising and safe therapeutic option against fast-evolving SARS-CoV-2 variants.

## 4 Discussion

The emergence and continuous spread of SARS-CoV-2 variants have raised substantial concerns regarding the efficacy of current antibody-based therapies against COVID-19 (30, 31). Therefore, the development of novel therapeutic approaches, especially those capable of targeting multiple crucial domains of the virus, is critical to combating novel or resurgent SARS-CoV-2 variants. In this study, we addressed this challenge by introducing a dual-targeting strategy in the form of a bsAb capable of specifically recognizing both the SARS-CoV-2 RBD and FP, which are essential domains for viral attachment to the host cell membrane and fusion in SARS-CoV-2 infection. Extensive characterization and functional evaluation of our engineered IgG4 (S228P)-(scFv)_2_ bsAb, K203.A, revealed its strong ability to neutralize multiple SARS-CoV-2 variants, underscoring its therapeutic potential. Moreover, we also elucidated the usefulness of phage display technology, combined with an established recombinant antibody library, in the accelerated development of bsAbs against rapidly evolving SARS-CoV-2 variants. Conclusively, our study not only suggests the potential of phage display technology in rapidly generating targeted bsAb-based therapies but also provides a blueprint for designing bsAbs and incorporating a dual-targeting strategy in the development of next-generation antibody therapeutics for COVID-19.

To date, the development of SARS-CoV-2 neutralizing antibodies has primarily focused on the RBD within the S1 subunit of the spike protein, given its vital function in direct interaction with the human hACE2 receptor, which enables the virus to enter host cells (32, 33). However, the emergence of mutations, particularly within the RBD, has posed challenges to the efficacy of existing RBD-specific mAbs (34, 35). In contrast, the S2 subunit of the spike protein, including the FP region comprising amino acid residues 816–833, which are critical for mediating the fusion of the viral envelope with cellular membranes, has demonstrated a higher degree of conservation across SARS-CoV-2 variants and other betacoronaviruses (35, 36). This region’s conservation is advantageous as it reduces the likelihood of mutations that compromise the efficacy of FP-targeting mAbs (37). Similar parallels have been observed in HIV-1 studies, where the gp41 FP, a surface-exposed region, has been identified as a target for neutralizing mAbs (38). However, past attempts to develop HIV-1 FP-targeting mAbs have encountered challenges, primarily due to their relatively low neutralizing potency (39, 40). Similarly, studies investigating the SARS-CoV-2 FP-specific mAb, COV44-79, and our own study with K107.1 showed relatively lower or no statistically significant *in vitro* neutralizing potency against the wild-type and variants because the majority of circulating SARS-CoV-2 does not expose their FP regions, which reside in the S2 domain of the spike proteins (17). The differences in efficacy between COV44-79 and K107.1 may be attributed to their recognition of distinct epitopes within the FP, warranting further investigation. In response to these challenges, we designed K203.A to target both the RBD and FP of SARS-CoV-2, with the aim of combining potency and broad efficacy. According to our *in vitro* neutralization assays, K203.A exhibited enhanced neutralizing potency against SARS-CoV-2 variants, especially against the Beta and Gamma variants, compared to the individual parental mAbs that target either RBD or FP. To address the underlying mechanism of this enhanced neutralization efficacy, it’s crucial to understand the mechanism of SARS-CoV-2 infection. Previous studies have reported that SARS-CoV-2 undergoes host receptor-mediated endocytosis following its attachment to the host cell through the interaction between RBD and hACE2 (41). After endocytosis, the virus exposes its FP, facilitated by the host cell proteases in the endosomal compartment, initiating fusion pore formation and subsequent membrane fusion (42). It has been known that the Beta and Gamma variants of SARS-CoV-2 have a unique E484K mutation, also identified as an escape mutation, in the RBDs (43). Based on previous reports, this mutation induces a conformational change of the RBDs and their tighter binding to hACE2, which facilitates endocytosis and increases viral infectivity (44). Ultimately, the tight association induced by this mutation weakens the neutralizing efficacy of existing RBD-specific mAbs, including our K102.1 on the Beta and Gamma variants (45). Here, we propose the underlying mechanism of K203.A bsAb that offers enhanced neutralization. Inside the endosomal compartment, although like 102.1, the RBD-specific paratope in K203.A also has a weakened ability to inhibit the virus attachment to hACE2, additionally, the FP-specific paratope in K203.A intervenes and more effectively targets the exposed FP and inhibits the membrane fusion between viral and host cell membranes. This suggests that the bsAb’s dual-targeting strategy may provide enhanced viral inhibition by blocking viral attachment and fusion processes. In conclusion, our study highlights the significance of the FP region as a promising target for SARS-CoV-2 neutralizing antibodies, in addition to the well-established RBD target. The development of bsAbs targeting both the SARS-CoV-2 RBD and FP, as exemplified by K203.A, demonstrates the potential of dual-targeting bsAbs in overcoming challenges faced by individual mAbs, thereby providing a potential avenue for more effective therapeutic interventions against COVID-19.

In response to the ongoing emergence of new SARS-CoV-2 variants, the development of bsAbs targeting distinct regions within the spike protein represents a favorable strategy for managing COVID-19 (46–48). In this study, we introduced K203.A, a novel IgG4-based bsAb with several promising characteristics for therapeutic application. Using fully human mAbs in the preparation of K203.A reduces the risk of immunogenicity. Additionally, K203.A exhibited strong binding affinity in the low nanomolar range for both the SARS-CoV-2 RBD and FP, indicating its potential for specific *in vivo* targeting of the virus. The high thermal stability of K203.A, with a T_m_ of 72.03°C, suggests its potential resilience under physiological conditions. Notably, K203.A demonstrated potent neutralizing capabilities against the SARS-CoV-2 Delta variant in hACE2-TG mice, as evidenced by its efficacy at a low dose of 5 mg/kg when administered intravenously, which is a common clinical route for antibody drug administration. Furthermore, K203.A exhibited an *in vivo* half-life of 2.09 days in mice, which is comparable to FDA-approved antibodies with established clinical efficacy, such as trastuzumab and cetuximab (49, 50). This suggests that K203.A has the potential to maintain a therapeutic presence in the body similar to these clinically proven antibodies. Importantly, K203.A is based on the IgG4 subtype, which minimizes the effector function-related toxicity commonly associated with IgG1-based antibodies and reduces the risk of unwanted *in vivo* toxicity, such as antibody-dependent cell cytotoxicity and cell phagocytosis, and further improves its safety profile. In our study, K203.A neither exhibited significant *in vivo* toxicity nor showed the potential for ADE. These findings confirmed that the novel IgG4-based bsAb K203.A holds therapeutic potential against newly emerging or resurging SARS-CoV-2 variants.

The emergence and continuous spread of SARS-CoV-2 variants necessitate the urgent development of effective antibodies to combat COVID-19 (51). However, conventional methods of isolating SARS-CoV-2 neutralizing antibodies from the whole blood of recovered patients or immunized mice can be laborious and time-consuming (52–54). Therefore, our study introduces an alternative and expedited approach for generating antibodies that can target emerging or resurgent SARS-CoV-2 variants. Herein, we successfully generated a novel bsAb in an IgG4 (S228P)-(scFv)_2_ format. This bsAb was created by rapidly isolating SARS-CoV-2 FP-specific mAbs from an established human recombinant antibody library. These FP-specific mAbs were then combined with previously identified RBD-specific mAbs obtained through phage display technology. The resulting bsAb exhibited enhanced neutralizing activity against various SARS-CoV-2 variants, including wild-type, Alpha, Beta, Gamma, Delta, and Kappa, showing the potential of our phage display-derived bsAb and offering valuable insights for the accelerated development of such antibodies. Moreover, sequence comparison revealed no mutations within the FP (S816–F833) across all currently reported SARS-CoV-2 variants, including Omicron subvariants and SARS-CoV (**Supplementary Figure 8**). This complete conservation of the FP sequence suggests that antibody-based targeting of the FP may be effective against a broad range of SARS-CoV-2 variants and potentially other betacoronaviruses as well. Although the underlying mechanisms remain to be elucidated, we propose that our bispecific antibody platform could serve as a universally applicable strategy against both SARS-CoV-2 and its related strains by simply switching RBD-specific antibodies.

In conclusion, our study introduces a novel dual-targeting approach with IgG4-based bsAb K203.A, which targets both the SARS-CoV-2 RBD and the highly conserved FP. The successful development of K203.A and its outstanding neutralizing efficacies highlight its promise as an efficient antibody development technique by integrating the specificity and potency of two different antibody targets. By combining multiple targets in a single bsAb, K203.A represents a promising avenue for the timely and successful management of the continuously changing SARS-CoV-2 and potential future viruses. Importantly, the use of the IgG4 format in bsAb development reduces the risk of potential *in vivo* toxicity compared with conventional IgG1-based virus-neutralizing antibodies, further enhancing its safety profile. In addition, our study highlights the potential of phage display-derived antibodies as a rapid and efficient method for generating bsAbs against the continuously evolving SARS-CoV-2. This approach offers a valuable tool for responding to emerging viral variants quickly and effectively. In summary, the insights gained from this study contribute to ongoing efforts to rapidly develop effective antibodies, addressing the constantly evolving landscape of infectious diseases. We believe that this novel approach represents a significant step forward in the field of antibody-based therapies and has the potential to make a substantial impact on managing current and future viral threats. In the near future, we aim to expand the antibody platform by developing bsAbs based on the Omicron variant RBD-specific mAbs isolated from our established human recombinant antibody library, thereby further demonstrating its utility in combating the current SARS-CoV-2 variants of concern.

## Data availability statement

The original contributions presented in the study are included in the article/**Supplementary Material**. Further inquiries can be directed to the corresponding author.

## Ethics statement

The animal study was approved by Institutional Animal Care and Use Committee, Institutional Biosafety Committee of Seoul National University Bundang Hospital. The study was conducted in accordance with the local legislation and institutional requirements.

## Author contributions

JK: Data curation, Formal Analysis, Investigation, Methodology, Visualization, Writing – original draft, Writing – review & editing. HK: Data curation, Formal Analysis, Investigation, Methodology, Visualization, Writing – original draft, Writing – review & editing. KH: Data curation, Investigation, Methodology, Visualization, Writing – review & editing. YL: Data curation, Investigation, Writing – original draft. HJ: Data curation, Investigation, Writing – original draft. HL: Data curation, Resources, Supervision, Writing – review & editing. JP: Data curation, Investigation, Writing – original draft. YC: Investigation, Writing – review & editing. JL: Investigation, Writing – review & editing. HS: Investigation, Writing – review & editing. HY: Investigation, Writing – review & editing. HC: Investigation, Writing – review & editing. HS: Resources, Writing – review & editing. SL: Conceptualization, Funding acquisition, Project administration, Resources, Supervision, Writing – original draft, Writing – review & editing.
