# Supplementary material for: A novel bispecific antibody dual-targeting approach for enhanced neutralization against fast-evolving SARS-CoV-2 variants
Source: Front Immunol. 2023 Sep 26;14:1271508. doi: 10.3389/fimmu.2023.1271508 (PMC10562541; doi:10.3389/fimmu.2023.1271508)
Supplement: Supplementary file 1 [file DataSheet_1.docx]

Supplementary Materials

**A Novel Bispecific Antibody Dual-Targeting Approach** **for Enhanced Neutralization Against Fast-Evolving SARS-CoV-2 Variants**

Ji Woong Kim^1,†^, Hyun Jung Kim^1,†^, Kyun Heo^1,2,3^, Yoonwoo Lee^4^, Hui Jeong Jang^4^, Ho-Young Lee^4^, Jun Won Park^5^, Yea Bin Cho^1^, Ji Hyun Lee^2^, Ha Gyeong Shin^2^, Ha Rim Yang^2^, Hye Lim Choi^2^, Hyun Bo Shim^6^ and Sukmook Lee^1,2,3,^*

^1^Department of Biochemistry, Kookmin University; Seoul 02707, Republic of Korea

^2^Department of Biopharmaceutical Chemistry, Kookmin University; Seoul 02707, Republic of Korea

^3^Antibody Research Institute, Kookmin University; Seoul 02707, Republic of Korea

^4^Department of Nuclear Medicine, Seoul National University Bundang Hospital; Seoul 13620, Republic of Korea

^5^Division of Biomedical Convergence, Kangwon National University; Chuncheon 24341, Republic of Korea

^6^Department of Life Sciences, Ewha Womans University; Seoul 03760, Republic of Korea

*** Correspondence:** Sukmook Lee; lees2018@kookmin.ac.kr
†These authors have contributed equally to this work


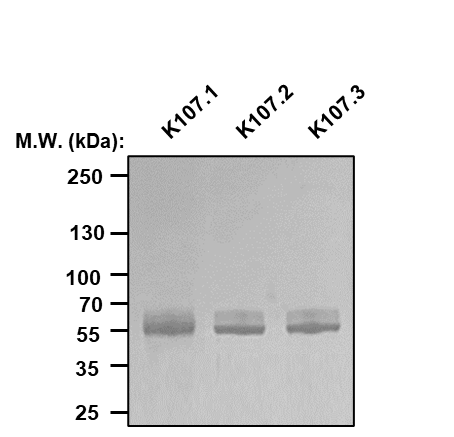


Supplementary Figure 1. The size and purity analysis of the purified FP-specific antibodies. Purified samples of scFv-Fc antibodies were subjected to 12% SDS-PAGE and stained with Coomassie Brilliant Blue, a common protein-staining dye used in electrophoresis. The molecular weights of protein standards are indicated on the left of each panel, serving as reference markers for determining the size of the purified antibodies. SDS-PAGE was conducted under reducing conditions, which is a common practice to denature proteins and dissociate any disulfide bonds, ensuring accurate separation based on size. Fusion peptide, FP; single-chain variable fragments, scFv; constant fragment, Fc; sodium dodecyl-sulfate polyacrylamide gel electrophoresis, SDS-PAGE.

Supplementary Figure 2. Production yield of purified fusion peptide-specific antibodies obtained from transient expression. The selected antibodies were transiently overexpressed. Following the expression process, purification was carried out using affinity column chromatography, a common method used to isolate specific proteins from complex mixtures. The bar graph presents the production yield of each scFv-Fc antibody. Single-chain variable fragments, scFv; constant fragment, Fc.


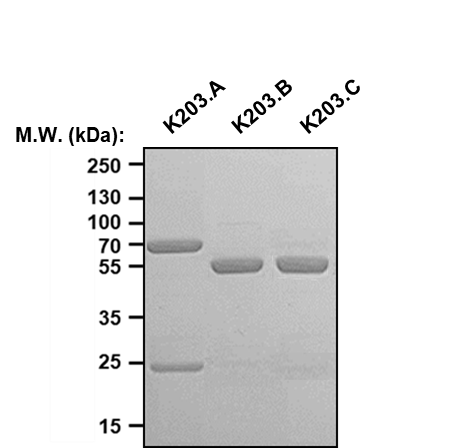


Supplementary Figure 3. The size and purity analysis of purified bsAbs. The purified bsAb samples were subjected to 12% SDS-PAGE and stained with Coomassie Brilliant Blue. The molecular weights of protein standards are indicated on the left side of each panel, serving as reference markers to determine the size of the purified bsAbs. SDS-PAGE was performed under reducing conditions, which allows for the denaturation of proteins and the disruption of disulfide bonds, facilitating accurate separation based on size. Bispecific antibody, bsAb; sodium dodecyl-sulfate polyacrylamide gel electrophoresis, SDS-PAGE.

Supplementary Figure 4. Evaluation of the thermal stability of K203.A. To assess thermal stability, K203.A was prepared at a concentration of 1 mg/mL in PBS with a pH of 7.4. The sample was then combined with Protein Thermal Shift^TM^ assay reagents, a technique commonly used to determine the T_m_ of proteins. The T_m_ of K203.A is determined by identifying the temperature at which the derivative peak occurs, indicating a significant change in the protein’s conformation and stability. Phosphate-buffered saline, PBS; melting temperature, T_m_.


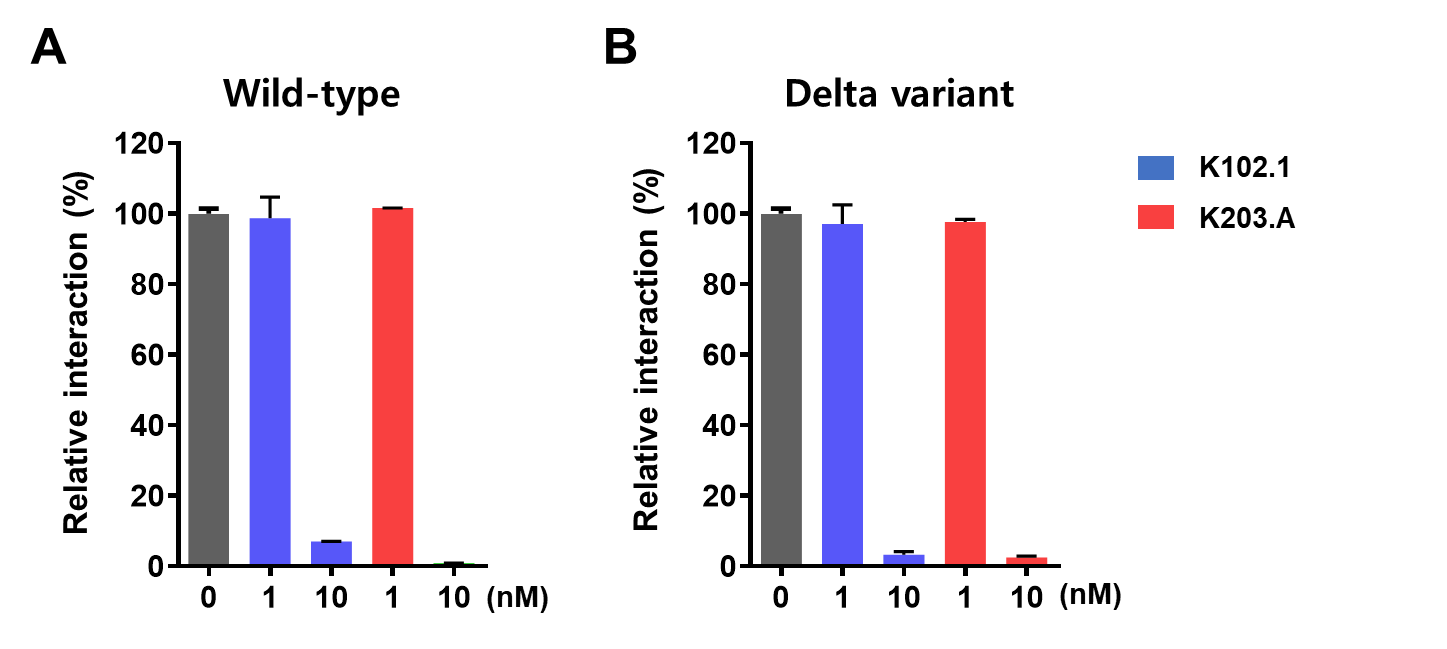


Supplementary Figure 5. Evaluation of the inhibitory effect of antibodies on the interaction between hACE2 and the RBDs of both the SARS-CoV-2 wild-type and the Delta variant. Inhibitory potency of K102.1 and K203.A at different concentrations on the interaction between hACE2 and SARS-CoV-2 wild-type (A) and Delta variant (B) RBDs were tested. Data are represented as mean ± SD of duplicates and represent one of two independent experiments. Human angiotensin-converting enzyme 2, hACE2; receptor-binding domain, RBD; standard deviation, SD.


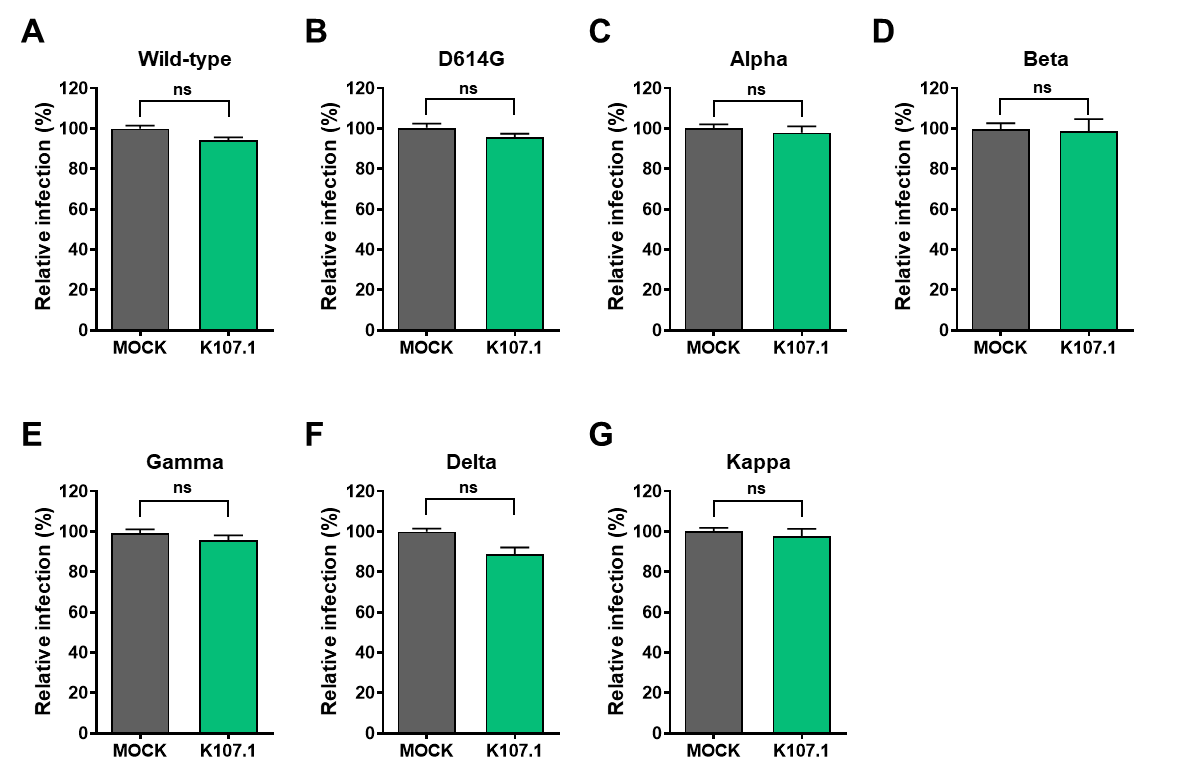


Supplementary Figure 6. Assessment of the neutralizing activity of fusion peptide-specific antibody K107.1 against pseudotyped SARS-CoV-2 wild-type and its variants. The neutralizing activity of K107.1 against SARS-CoV-2 variants, including the wild-type (A) and D614G (B), Alpha (C), Beta (D), Gamma (E), Delta (F), and Kappa (G) variants, was evaluated at a concentration of 200 nM. The neutralizing activity of K107.1 was assessed using duplicate measurements. The resulting values are represented as the mean ± SD. Standard deviation; SD, not significant, ns.


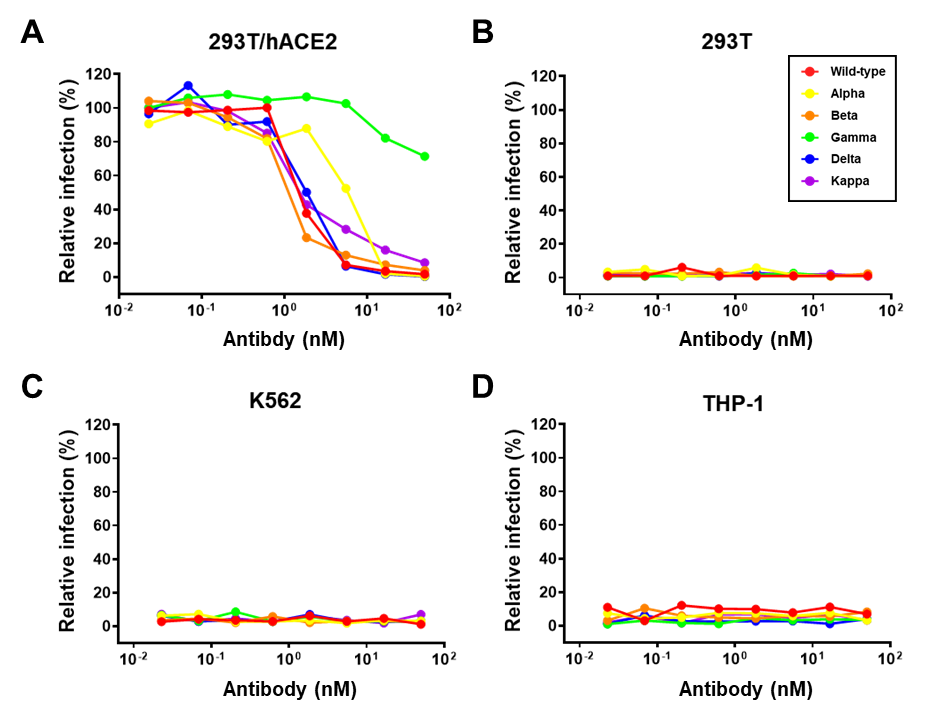


Supplementary Figure 7. Evaluation of the antibody-dependent enhancement activity of K203.A on Fc receptor-expressing cells. The ADE measurement was performed using SARS-CoV-2 pseudoviral infection of different cell types, including 293T/hACE2 (A), 293T (B), K562 (C), and THP-1 (D). Data are represented as mean ± SD of duplicates and are from one of two independent experiments. Constant fragment, Fc; antibody-dependent enhancement, ADE; standard deviation, SD.


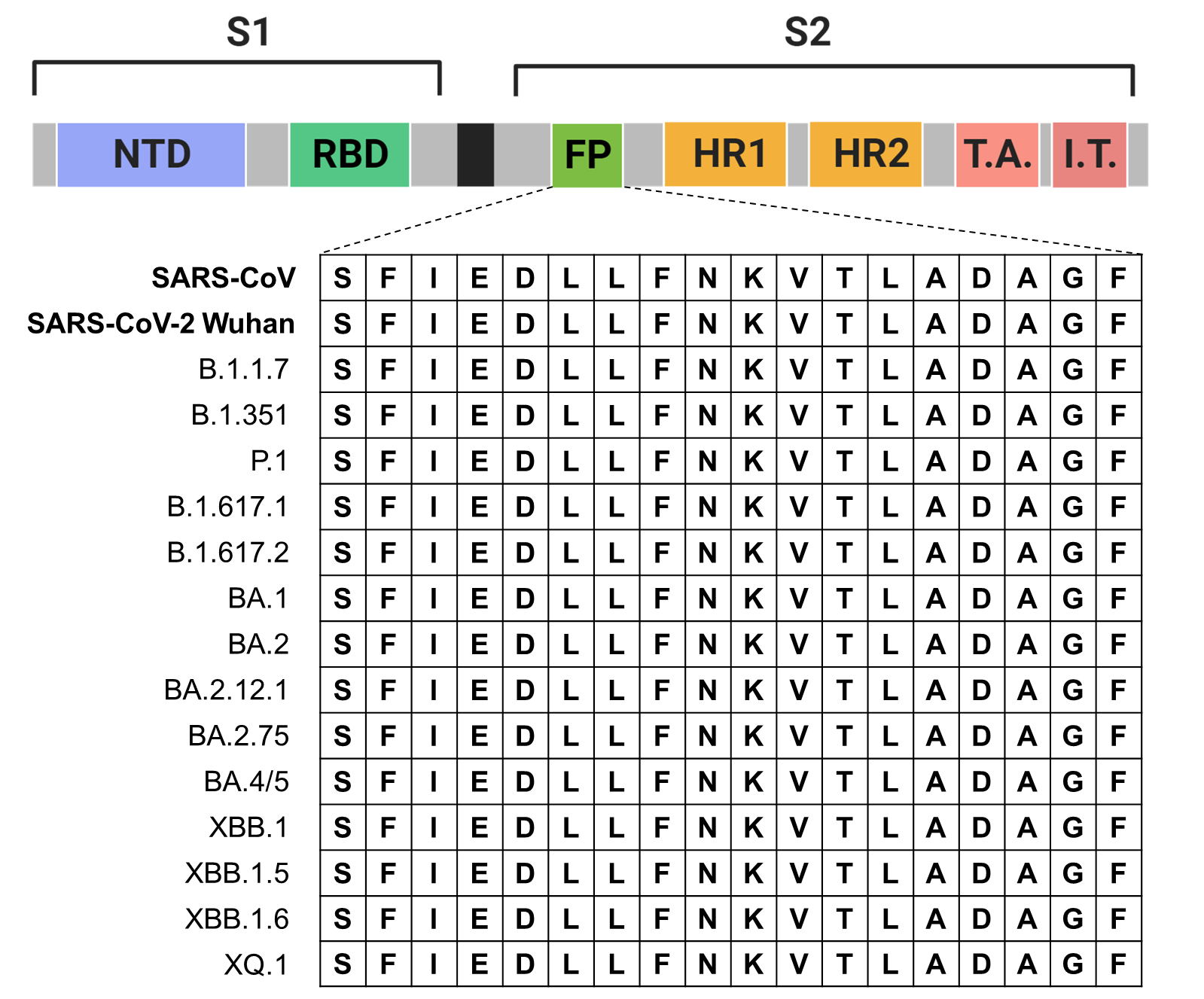


Supplementary Figure 8. Comparative analysis of fusion peptide sequences of SARS-CoV and SARS-CoV-2 variants. The figure provides a comprehensive sequence alignment of the fusion peptide sequences of SARS-CoV and various of SARS-CoV-2 variants. Single-letter abbreviations for amino acids: A, Ala; D, Asp; E, Glu; F, Phe; G, Gly; I, Ile; K, Lys; L, Leu; N, Asn; S, Ser; T, Thr; and V, Val.
